# Coupling relationships between vegetation and soil in different vegetation types in the Ulan Buh Desert and the Kubuqi Desert

**DOI:** 10.3389/fpls.2025.1505526

**Published:** 2025-03-13

**Authors:** Gaoling Han, Jianqiang Huo, Rui Hu, Xiangwen Gong, Yicong Nan, Yuchao Lian, Zhishan Zhang

**Affiliations:** ^1^ Key Laboratory of Ecological Safety and Sustainable Development in Arid Lands/Shapotou Desert Research and Experiment Station, Northwest Institute of Eco-Environment and Resources, Chinese Academy of Sciences, Lanzhou, China; ^2^ University of Chinese Academy of Sciences, Beijing, China

**Keywords:** revegetation, natural vegetation, vegetation-soil system, coupling coordination, revegetation construction

## Abstract

**Introduction:**

Desertification is a globally recognized ecological issue that poses severe threats to the environment, economic and social systems. Revegetation is the primary means to combat desertification, yet the effectiveness of revegetation practices requires reasonable quantification.

**Methods:**

To identify appropriate planting patterns for revegetation in different deserts and provide a basis for vegetation reconstruction in deserts, we conducted a comprehensive survey in the Ulan Buh Desert and the Kubuqi Desert of the Northern China. Data on vegetation and soil were collected from 54 representative sites, covering both natural and revegetation communities.

**Results:**

The findings revealed that the diversity of herbaceous and woody species, and soil nutrient content increased after revegetation, in comparison to adjacent moving sand dunes. Additionally, the species diversity and soil conditions in revegetation areas, gradually approached those of natural vegetation communities, indicating a succession towards a state resembling natural conditions. Variations in the coupling of vegetation-soil systems were observed among different community types in both deserts. Notably, the communities dominated by *Caragana korshinskii* and *Artemisia ordosica* exhibited the strongest coupling in the vegetation-soil system, driven primarily by soil water and nutrients, as well as vegetation growth.

**Discussion:**

Evaluation of vegetation-soil system coupling effect was used to evaluate the effectiveness of vegetation restoration and species selection in the wo deserts, which can serve as a reference for vegetation reconstruction and ecological restoration in desert areas.

## 1 Introduction

Desertification is an increasingly severe ecological problem worldwide due to climate change, soil erosion, natural disasters, and unreasonable human activities (Cao et al., 2009; Yang and Wu, 2010; Odjugo and Isi, 2003; Kar, 2018; Meng et al., 2021). The vegetation-soil system is a dynamic equilibrium ecosystem based on the reciprocal effects of vegetation, soil, and the complex biogeochemical process (Zeng et al., 2006; Chi et al., 2022). Vegetation-soil system coupling refers to the interactive and interdependent relationship between vegetation and soil (Wang et al., 2021). This coupling is fundamental to the functions and services of ecosystems and is of great significance for the study of ecological restoration (Ma et al., 2023). Desertification not only causes the vegetation destruction and soil degradation, but also weakens the coupling effects between vegetation-soil systems (Fu et al., 2011). China is also influenced by desertification, which exerts significant impacts on ecological, economic, and social systems (Wang et al., 2002; Bao et al., 2017; Zhang and Huisingh, 2018). To combat desertification, China has initiated a series of key ecological projects (He et al., 2015; Hu et al., 2021; Zhai et al., 2023). Numerous studies have focused on the ecological effects of these projects and paid excessive attention to the impacts of isolated indicators like vegetation characteristics, while overlooking a comprehensive assessment that includes the ecosystem health, biodiversity, and soil quality (Zhang et al., 2016; Dou et al., 2024).

In desert ecosystems, vegetation restoration plays a significant role in reducing wind erosion and stabilizing the mobility of sandy soils. Consequently, selecting appropriate plant species is crucial during the process of vegetation restoration (Zhang et al., 2016). In order to scientifically quantify the effectiveness of revegetation, it is necessary to set the adjacent natural communities and highly degraded moving sand dunes as control. The purpose of this approach is to assess the ecological restoration effectiveness of revegetation on the structure of vegetation and soil properties (Li et al., 2018; Zhou et al., 2023). Building on this foundation, the present study will conduct a scientific quantitative analysis of the ecological restoration effects of vegetation restoration in improving vegetation coverage and soil properties, using adjacent natural plant communities and highly degraded moving sand dunes as controls.

The common method assessing the effects of revegetation is to compare changes in vegetation cover, species diversity, soil properties after revegetation (Courtney et al., 2009). But rarely consider the coupling effects between them (Zhao et al., 2022). The traditional methods tend to consider vegetation and soil properties separately (Pan et al., 2019), but rarely consider the coupling effects between them (Zhao et al., 2022). Separate analysis of vegetation and soil considers their respective characteristics and changes individually, whereas vegetation-soil system coupling analysis takes into account the interactions between the two subsystems, enabling more accurate predictions of the ecosystem’s response to environmental changes. The vegetation-soil system coupling evaluates the restoration status of both vegetation and soil (Li et al., 2020; Zhang et al., 2013). Likewise, vegetation-soil coupling models offer theoretical foundations for ecological restoration (Du et al., 2013; Wang et al., 2021; Puyang et al., 2021). Therefore, understanding evaluation of vegetation-soil system coupling effect in desert ecosystems provides scientific evidences for species selection and configuration, and further ensures the sustainability of ecological restoration.

This study selected the Wulan Buhe Desert and the Kubuqi Desert as the research areas, with survey points chosen based on the different vegetation types in the two deserts. These two deserts exhibit significant spatiotemporal heterogeneity in terms of geographical location, climatic conditions, dune morphology, vegetation coverage, and the impacts of human activities (Zhu et al., 2025). Additionally, different regions of the two deserts are characterized by various types of vegetation restoration. Therefore, different vegetation restoration models should be adopted for revegetation construction and ecological restoration in these two deserts. Effect value is used to quantify the magnitude of the effect of an experiment or intervention, describing the average difference between two or more groups on a specific variable. In this study, we compared the effect values of revegetation with natural vegetation and moving sand dunes in the Ulan Buh Desert and Kubuqi Desert to investigate the influence of revegetation on species diversity and soil nutrient content. Furthermore, we conducted an investigation into the vegetation and soil characteristics of different vegetation types in the two deserts and explored appropriate strategies for vegetation restoration, with the aim of determining the most suitable replanting patterns for these two deserts. To achieve these objectives, we posited the following hypotheses: 1) Compared to the adjacent moving sand dunes, revegetation would improve the plant and soil conditions, which were similar to the adjacent natural vegetation community. 2) Different community types will exhibit varying degrees of vegetation-soil system coupling, and the extent of recovery of vegetation and soil will differ following the planting of different revegetation types.

## 2 Materials and methods

### 2.1 The study area

**Figure 1:**
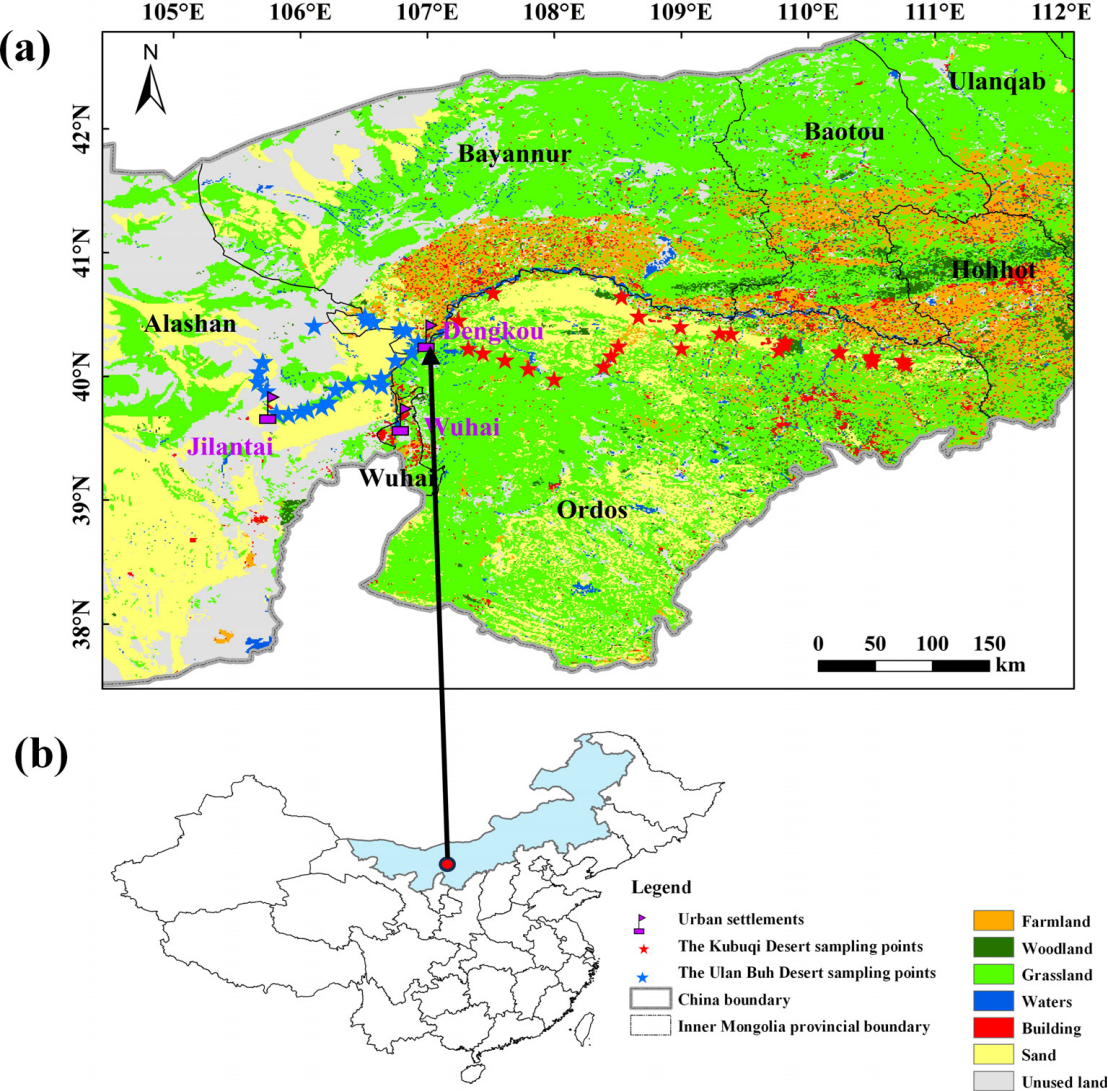
Location of the study area and distribution of the sampling points. **(a)** Distribution of sampling points in the Ulan Buh Desert and Kubuqi Deserts **(b)** Location of research areas on the map of China.

The study area is located at the critical area for mitigating desertification in the Ulan Buh Desert and Kubuqi Deserts. The Ulan Buh Desert is located in the middle reaches of the Yellow River and belongs to a transitional zone from warm-temperate semi-arid to arid climate (Li et al., 2018). The annual average precipitation is about 140 mm, and the annual average evaporation ranges from 2110 to 2995 mm (Li et al., 2024). The predominant soil type is aeolian sand (Tian et al., 2019), and the vegetation is dominated by xerophytic herbaceous plants and shrubs. The Kubuqi Desert is located at the northern edge of the Ordos Plateau, falling within the temperate arid and semi-arid regions. The annual precipitation is about 249 mm, and the annual average evaporation ranges from 2100 to 2700 mm (Yang et al., 2016; Chen et al., 2022). The predominant soil types are aeolian sand and gravelly sand (Dong et al., 2020; Sun et al., 2023), and the vegetation consists of steppe-like desert vegetation dominated by perennial grasses and small shrubs (**Figure 1**).

### 2.2 Vegetation survey, soil samples collection and measurement

We collected data from 24 survey plots in the Ulan Buh Desert and from 30 survey plots in the Kubuqi Desert from July and August in 2022 and 2023 (**Figure 1**). In the Ulan Buh Desert, the main survey line was established along the east-west sand-crossing highway from Jilantai to Wuhai, where ten survey points were set up. An additional line extended from Jilantai through Dengkou to Wuhai, with fourteen survey points established along this route. In the Kubuqi Desert, the main survey line was laid out along the east-west direction of the Expressway, with branch lines set up in the north-south direction. The north-south branch line was divided into three regions: east, middle, and west, with 11, 7, and 12 survey points, respectively. At each survey point, we established a transect line of 100 to 200 meters. In each survey site, a nested design was implemented along the transect line, consisting of three 10 m × 10 m quadrats for moving sand dunes, six for revegetation, and three for natural woody vegetation. Within each of these larger woody vegetation quadrats, a smaller 0.5 m × 0.5 m quadrat was nested for the purpose of studying herbaceous vegetation. When survey points were distant from moving sand dunes or lacked revegetation, only natural vegetation plots were established. In the Ulan Buh Desert, a total of 99 quadrats were established (N=33 for revegetation, 57 for natural vegetation, and nine in moving sand dunes), and in the the Kubuqi Desert, a total of 185 quadrats were established (98 for revegetation, 60 for natural vegetation, and 27 in moving sand dunes) (**Figure 1**; **Supplementary Figure S1**).

In each woody vegetation quadrat, we recorded the species, density and cover of woody plants, as well as the planting methods, row spacing (to evaluate the initial density of revegetation), and types of revegetation. In each herbaceous quadrat, we counted the number of species and estimated the vegetation cover. The above-ground biomass was collected using envelops in each herbaceous quadrat, and then was dried at 80°C in the laboratory for 48 hours, finally was weighed. The above-ground biomass was collected using envelops in each herbaceous quadrat, and then was dried at 80°C in the laboratory for 48 hours, finally was weighed. For both woody and herbaceous quadrats, we measured species richness (R) and calculated the Shannon-Wiener index (H’) and Simpson index (D) (Whittaker, 1972; Magurran and McGill, 2010; Tuomisto, 2010).

We collected latitude, longitude and altitude data of each sample plot using a GPS device. Meteorological data for the local area was obtained from the spatial interpolation dataset of China’s average meteorological elements using ArcGIS Desktop 10.8 software, based on the latitude and longitude of the sample plots (Spatial Interpolation Dataset of Average Conditions of Chinese Meteorological Elements: https://www.resdc.cn/DOI/DOI.aspx?DOIID=39). The acquired meteorological data mainly includes annual evaporation, annual average ground temperature, annual precipitation, annual average atmospheric pressure, annual average relative humidity, annual sunshine hours, annual average temperature, and annual average wind speed. We collected soil samples at nine depths (0, 0.1, 0.2, 0.4, 0.6, 0.8, 1.0, 1.2, 1.5 m) in the center of each woody quadrat. We placed the fresh soil into sealed bags and weighed them *in situ*. The soil samples were dried at 105°C for 24 hours and were weighed to get their dry weight in the laboratory. The gravimetric soil water content at different soil depths could be calculated. We collected surface (0-5 cm) soil samples using a 1×10^-4^ m^3^ core in each quadrat and measured soil bulk density in the laboratory. To measure soil saturated water content, we soaked the soil samples in water for 24 hours and then weighed them before and after drying at 105°C for 24 hours. We collected soil samples from 0-20 cm using a 1×10^-4^ m^3^ cutting ring and then divided them into two parts after air-drying. One part was passed through a 2 mm mesh sieve for measuring soil mechanical composition using the wet sieve and pipette method. The other part was passed through a 1 mm sieve to measure soil organic carbon, total carbon, total nitrogen, total phosphorus content, pH and conductivity. We tested the pH of a solution with a soil to water ratio of 1: 2.5 (g/ml) using a pH meter, and measured the electric conductivity of a solution with a soil to water ratio of 1:5 (g/ml) using a conductivity meter. Total carbon, total nitrogen, and total phosphorus contents were measured using an external heating-potassium dichromate method, a semi-micro Kjeldahl method, a sodium hydroxide melting-molybdenum antimony anti-colorimetric method, respectively (Pal, 2013; Haluschak, 2006).

### 2.3 Data analysis

#### 2.3.1 Comparison of vegetation and soil characteristics

We calculated effect values (Ln RR) of vegetation and soil indicators (ratios of indicators in revegetation to those in natural vegetation, and ratios of indicators in revegetation to those in moving sand dunes) as follows (Equation 1; Hedges et al., 1999):


(1) Ln RR=Ln (Xt/Xc)=Ln (Xt)−Ln (Xc)

Where Xc represents the value of the indicator of the revegetation community, Xt represents the indicator of the natural vegetation community or moving sand dunes.

We used one-way ANOVA to test the differences in vegetation and soil indicators among different community types, and used the Duncan test for multiple comparisons (α=0.05).

#### 2.3.2 Evaluation of vegetation-soil system coupling

The selection of indicators for vegetation-soil system coupling evaluation followed the principle of representativeness, scientificalness and independence. The evaluation system for vegetation-soil system with different vegetation community types in Ulan Buh Desert and Kubuqi Desert was divided into a vegetation subsystem and a soil subsystem, here in seven indicators were selected in each subsystem, after removing strongly correlated indicators (**Supplementary Figure S2**). The vegetation subsystem was comprised of herbaceous and woody indicator. The herbaceous indicator selected included herbaceous abundance, richness, coverage and biomass, as well as the woody abundance, richness and coverage. The soil subsystem consisted of soil physical property indicator and soil nutrient indicators, the former included soil water content, sand, silt and clay contents; the latter included organic carbon, total carbon and total phosphorus. Finally, we employed the entropy weight method (Zou et al., 2006) to determine the weights of vegetation and soil indicators and constructed an evaluation indicator system for vegetation-soil coupling (**Table 1**).

**Table 1:** Evaluation indicator system and indicator weight of vegetation-soil coupling.

Goal layer	Criterion layer	indicator layer				Comprehensive weight
		First grade indicators	Weight	Second grade indicators	Weight	
Evaluation of vegetation-soil coupling coordination degree	Vegetation comprehensive evaluation function (CCE)	Herbaceous indicator	0.5	Abundance	0.3758	0.1879
				Richness	0.1388	0.0694
				Coverage	0.2183	0.1091
				Biomass	0.2671	0.1336
		Woody indicator	0.5	Abundance	0.4159	0.2080
				Richness	0.1539	0.0770
				Coverage	0.4302	0.2151
	Soil comprehensive evaluation function (PCE)	Soil physical property indicator	0.75	Soil water content	0.2205	0.1329
				Sand	0.3252	0.0690
				Silt	0.3764	0.0481
				Clay	0.0780	0.2439
		Soil nutrient indicator	0.25	Organic carbon	0.5315	0.2823
				Total carbon	0.2762	0.0585
				Total phosphorus	0.1923	0.1653

We standardized the vegetation and soil indicators (Equation 2; Zeng et al., 2006; Guo et al., 2021) using the following formula:


(2) $$\text{ Ni}=\{\begin{array}{c} \frac{x_{i}-\min\left(x_{i}\right)}{\;\max\left(x_{i}\right)-\min\left(x_{i}\right)}\;\;(Positiveeffect）\\ \frac{\max\left(x_{i}\right)-x_{i}}{\max\left(x_{i}\right)-\min\left(x_{i}\right)}\;\;\;\;\;\;(Negativeeffect） \end{array}$$

Where Ni is the standardized value of each indicator, x_i_ is the value of the different indicators. Positive indicators included herbaceous abundance, richness, coverage, and biomass, woody abundance, richness, and cover, soil water content, organic carbon, total carbon, total phosphorus, clay and silt content. Negative indicators included sand content.

We calculated a comprehensive evaluation indicator of vegetation and soil as follows (Equations 3, 4; Zeng et al., 2006; Peng et al., 2011; Zhang et al., 2013; Zhao et al., 2022):


(3) $$\text{CCE}(x)=\sum_{i=1}^{n}p_{i}x_{i}$$

(4) $$\text{PCE}(y)=\sum_{i=1}^{n}q_{i}y_{i}$$

Where CCE is the vegetation evaluation indicator; PCE is the soil evaluation indicator; p_i_ and x_i_ are the weights and standardized values corresponding to the i-th indicator in the vegetation evaluation function, respectively; q_i_ and y_i_ are the weights and standardized values corresponding to the i-th indicator in the soil evaluation function, respectively. Higher CCE and PCE values indicate better vegetation growth status or soil properties.

We calculated the coupling coordination degree (Cd) of the vegetation-soil system as follows (Equations 5–7; Zeng et al., 2006; Peng et al., 2011; Zhang et al., 2013):


(5) $${\text{C}}_{d}=\sqrt{\text{C}\cdot \text{T}}$$

(6) T=aCCE(x)+bPCE(y)

(7) $$\text{C}=\sqrt{\frac{\text{CCE}\left(\text{x}\right)\cdot \text{PCE}\left(\text{y}\right)}{{\left(\text{CCE}\left(\text{x}\right)+\text{PCE}\left(\text{y}\right)\right)}^{2}}}$$

Where C_d_ is the coupling coordination degree of vegetation-soil system, which ranges between 0 and 1. The greater the C_d_ value, the more harmonious the coupling relationship of vegetation-soil system. T is the comprehensive harmonic indicator of vegetation-soil system, which reflects the synergistic effect of vegetation-soil system. C is the coupling degree of the vegetation-soil system, which ranges between 0 and 1, where C approaches 1, the relationship between vegetation and soil is more benign. a and b are coefficients of the CCE and PCE comprehensive evaluation functions, respectively. Because vegetation and soil both play important roles in ecological restoration, we set a and b both to 0.5. Finally, we used the coupling results to determine a reasonable vegetation construction mode (**Table 1**).

We used redundancy analysis (RDA) to analyze the factors that influence the coupling indicator, which we analyzed and plotted with Canoco5.

## 3 Results

### 3.1 The effect values of vegetation and soil indicators

**Figure 2:**
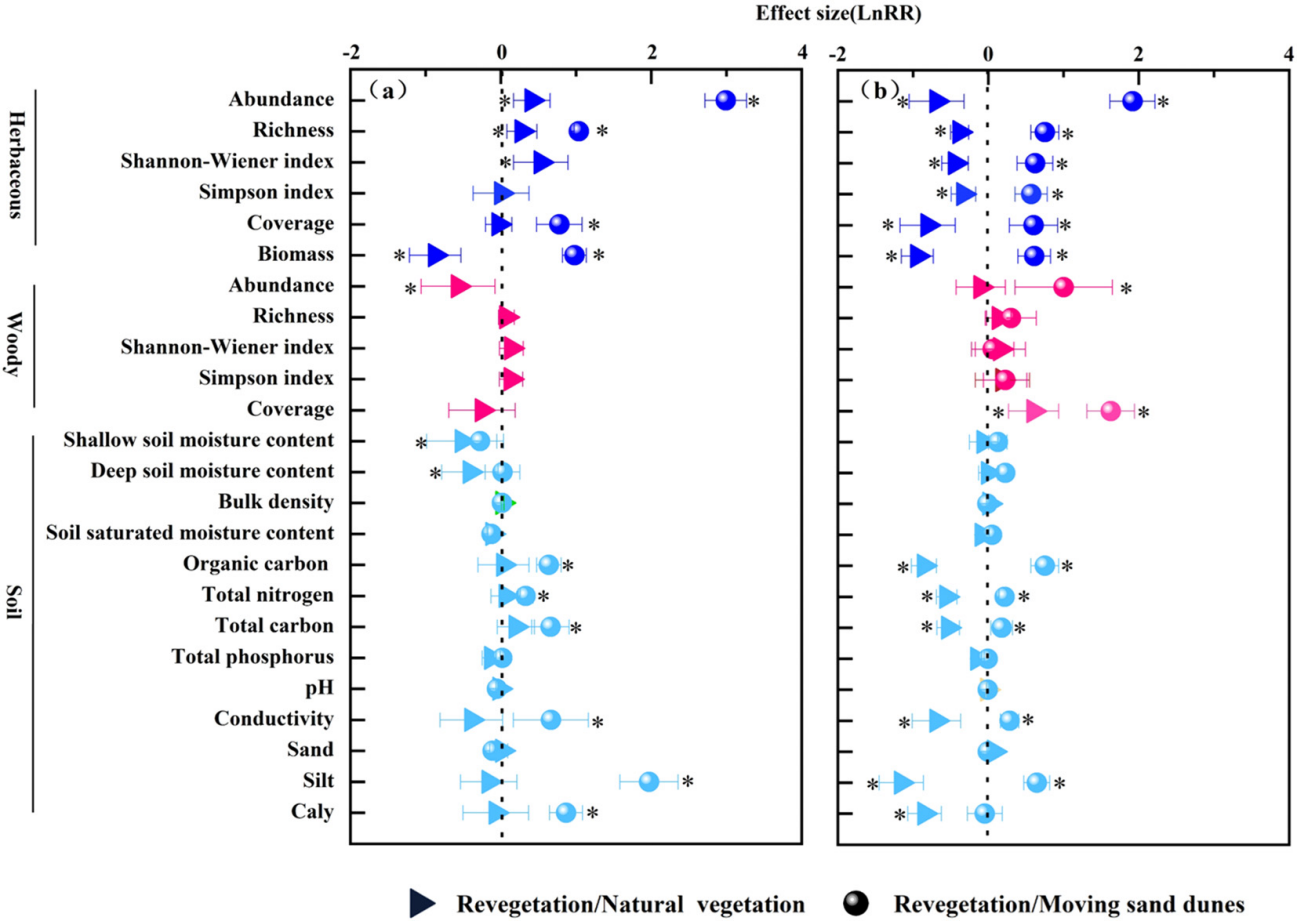
Vegetation and soil indicator effect values of different community types in Ulan Buh Desert **(a)** and Kubuqi Desert **(b)**. * represents significant effect size at p=0.05 level. (Effect value>0 indicates an increase in the indicator, and effect value<0 indicates a decrease in the indicator).

In the Ulan Buh Desert, we found that revegetation led to an increase in the abundance, diversity and richness of herbaceous species, but a decrease in herbaceous biomass and woody abundance compared to natural vegetation. Similarly, compared to moving sand dunes, revegetation resulted in higher herbaceous abundance, richness, coverage and biomass; in addition, the soil water content in both shallow (0-0.3 m) and deep (0.4-1.5 m) layers decreased, while soil organic carbon, total nitrogen, total carbon, silt, clay, and electrical conductivity increased in both natural and revegetation communities (**Figure 2**).

In the Kubuqi Desert, revegetation led to lower herbaceous abundance, richness, diversity cover, and biomass, but higher woody cover compared to the natural vegetation. There were higher herbaceous abundance, richness, and diversity, as well as higher woody abundance and cover in revegetated areas compared to moving sand dunes. In revegetated areas, the soil organic carbon, total nitrogen, total carbon, silt, clay contents, and electrical conductivity were lower than those in natural vegetation areas, while the organic carbon, total nitrogen, total carbon, clay content, and electrical conductivity were higher than those in moving sand dunes (**Figure 2**).

### 3.2 Vegetation-soil system coupling of different community types in two deserts

We found that the natural *Caragana korshinskii* community, the *Artemisia ordosica* community and the *Nitraria tangutorum*-*Haloxylon ammodendron* community exhibited stronger vegetation-soil system coupling among the natural vegetation communities in the Ulan Buh Desert, and the coupling degree values (C) were 0.496, 0.492 and 0.486, respectively. Likewise, the natural *Kalidium foliatum* community, *N. tangutorum* community and *N. tangutorum*-*H. ammodendron* community exhibited better vegetation-soil system coupling and coordination. Their respective coupling coordination degree values (C_d_) were 0.594, 0.515, and 0.471. In the revegetation community, the vegetation-soil system coupling was highest in the *H. ammodendron*, followed by the *Hedysarum scoparium* community, and the worst in the *C. korshinskii* community. Their respective C values were 0.495, 0.469 and 0.440. The vegetation-soil system coupling coordination state was highest in the *C. korshinskii* and *H. ammodendron* communities, but lowest in the *H. scoparium*, their C_d_ values were 0.494, 0.412, and 0.324, respectively. Compared to the natural *C. korshinskii* community, the coupling relationship between vegetation and soil of the revegetation *C. korshinskii* community was improved, and the vegetation-soil system also showed a good coupling state (**Table 2**).

**Table 2:** Coordination of vegetation-soil system coupling in different desert community types.

Desert	Area	Community category	CCE	PCE	C	T	C_d_	CCE/PCE
Ulan Buh Desert	Natural	Pha	0.380	0.135	0.440	0.258	0.337	2.820
		Nit	0.260	1.082	0.395	0.671	0.515	0.240
		Kaf	0.308	1.613	0.367	0.960	0.594	0.191
		Amm	0.259	0.596	0.460	0.428	0.443	0.435
		Aro	0.259	0.372	0.492	0.315	0.394	0.695
		Cos	0.557	0.211	0.447	0.384	0.414	2.635
		Aro-Ars	0.251	0.076	0.423	0.164	0.263	3.293
		Nit- Haa	0.350	0.563	0.486	0.457	0.471	0.621
		Cak	0.436	0.333	0.496	0.384	0.436	1.309
	Revegetation	Haa	0.296	0.389	0.495	0.342	0.412	0.759
		Cos	0.302	0.147	0.469	0.224	0.324	2.055
		Cak	0.292	0.817	0.440	0.554	0.494	0.357
	Moving sand dunes	Moving sand dune	0.004	0.159	0.153	0.082	0.112	0.025
Kubuqi Desert	Natural	Aro-Nit	0.557	1.012	0.479	0.784	0.613	0.551
		Aro	0.828	0.692	0.498	0.760	0.615	1.197
		Krc	0.527	0.960	0.478	0.744	0.596	0.549
		Cab	0.836	0.702	0.498	0.769	0.619	1.191
		Cat	0.907	1.023	0.499	0.965	0.694	0.886
		Nit-Tac	1.021	1.260	0.497	1.140	0.753	0.811
		Nes	0.939	0.920	0.500	0.930	0.682	1.021
		Aro-Cof	1.005	0.287	0.416	0.646	0.518	3.498
		Cof	0.751	0.429	0.481	0.590	0.533	1.750
	Revegetation	Cof	0.739	0.284	0.448	0.511	0.478	2.606
		Haa	0.277	0.417	0.490	0.347	0.412	0.664
		Cam	0.532	0.162	0.423	0.347	0.383	3.279
		Sac	0.559	0.276	0.471	0.418	0.443	2.024
		Aro	1.140	1.192	0.500	1.166	0.763	0.956
		Pop	0.594	0.369	0.486	0.481	0.484	1.608
		Pop-Sac	0.256	0.172	0.490	0.214	0.324	1.485
		Cak	0.824	0.675	0.498	0.750	0.611	1.220
	Moving sand dunes	Moving sand dunes	0.145	0.231	0.487	0.188	0.302	0.626

Pha, *Phragmites australis*; Nit, *Nitraria tangutorum*; Kaf, *Kalidium foliatum*; Amm, *Ammopiptanthus mongolicus*; Aro, *Artemisia ordosica*; Cos, *Corethrodendron scoparium*; Ars, *Artemisia sieversiana*; Haa, *Haloxylon ammodendron*; Cak, *Caragana korshinskii*; Krc, *Krascheninnikovia ceratoides*; Cab, *Caragana brachypoda*; Cat, *Caragana tibetica*; Tac, *Tamarix chinensis*; Nes, *Neotrinia splendens*; Cof, *Corethrodendron fruticosum*; Cam, *Calligonum mongolicum*; Sac, *Salix cheilophila*; Pop, *Populus przewalskii*.

The coupling between vegetation communities and soil was higher in natural vegetation communities of the Kubuqi Desert. This included higher C values of natural *Achnatherum* sp*lendens*, *Caragana tibetana*, and *A. ordosica* communities were 0.500, 0.499 and 0.498, respectively. Likewise, the natural *N. tangutorum*-*Tamarix chinensis*, *C. tibetana* and *A. ordosica* communities had higher coupling coordination states of the vegetation-soil system, with high C_d_ values of 0.753, 0.694 and 0.682 respectively (**Table 2**). In revegetated areas, the vegetation-soil system coupling in the *A. ordosica*, *C. korshinskii*, and *Populus przewalskii-Salix psammophila* revegetation communities was relatively higher, and the C values of them were 0.500, 0.498, and 0.490, respectively. The revegetated communities of *A. ordosica*, *C. tibetana* and *P. przewalsk*ii had the relatively higher vegetation-soil system coupling coordination states, with C_d_ values of 0.763, 0.612, and 0.484, respectively. The vegetation-soil system coupling and its coordination states of revegetated *A. ordosic*a community were higher than those of natural *A. ordosica* community (**Table 2**).

**Figure 3:**
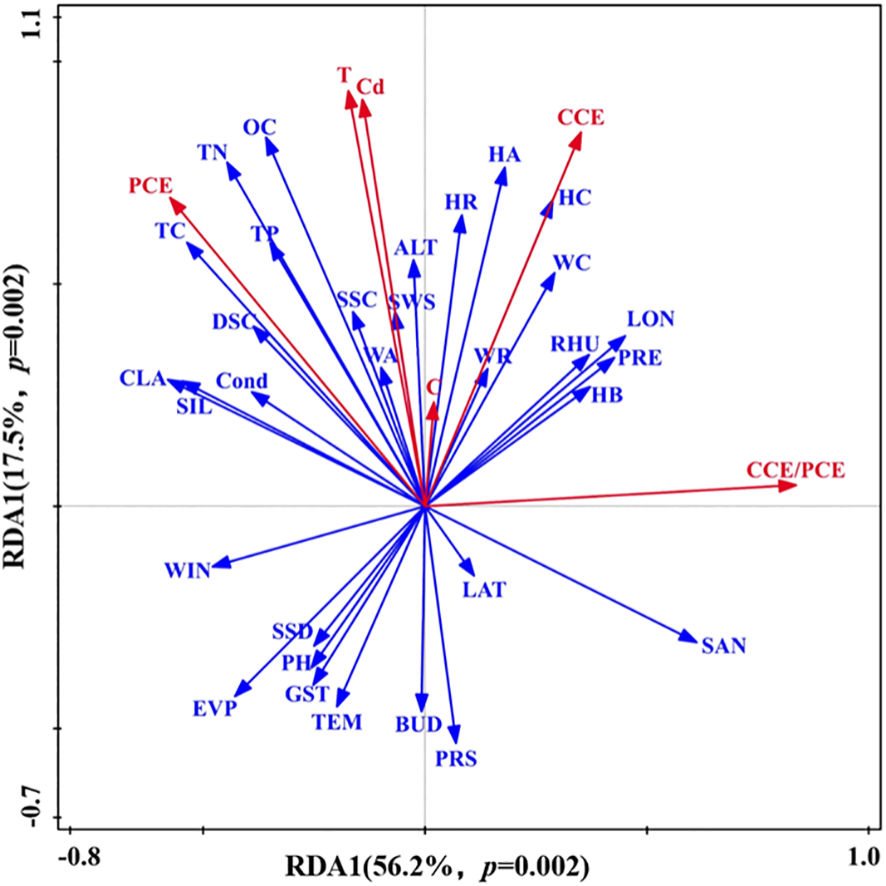
Redundancy analysis of vegetation-soil system coupling results and environmental factors. LON, Longitude; LAT, Latitude; ALT, Altitude(m); EVP, Annual evaporation(mm); GST, Average annual ground temperature(°C); PRE, Annual precipitation(mm); PRS, Annual mean air pressure(hPa); RHU, Average annual relative humidity(%); SSD, Sunshine hours per year(h); TEM, Average annual temperature(°C); WIN, Average annual wind speed(m/s); OC, Organic carbon(g/kg); TN, Total nitrogen(g/kg); TC, Total carbon(g/kg); TP, Total phosphorus(g/kg); Cond, Conductivity(us/cm); SAN, Sand(%); SIL, Silt(%); CLA, Clay(%); BUD, Bulk density(g/cm3); SWS, Soil saturated water content(%); HA, Herbaceous abundance; HR, Herbaceous richness; HC, Herbaceous coverage(%); HB, Herbaceous biomass(g/m2); WA, Woody abundance; WR, Woody richness; WC, Woody coverage(%); SSC, Shallow soil moisture content; DSC, Deep soil moisture content.

We found that the first axis (RDA1) and the second axis (RDA2) explained 56.2% and 17.5% of the influence of environmental factors on the vegetation-soil system coupling, respectively. Specifically, we found that vegetation-soil system had strong correlations with total nitrogen, total carbon, the cover, abundance, richness, and biomass of herbaceous species, as well as woody cover and richness, while it was negatively correlated with soil pH, sand content, wind speed and temperature. Furthermore, water status, vegetation growth and soil nutrient contents also influence the vegetation-soil system coupling (**Figure 3**).

### 3.3 Identification of optimal revegetation species in Ulan Buh Desert and Kubuqi Desert

We compared vegetation and soil indicators, and vegetation-soil system coupling to select the proper revegetation species and configurations. In the Ulan Buh Desert, compared to the natural *C. korshinskii* community, the woody abundance, richness, and coverage of revegetated *C. korshinskii* community increased by 2.90%, 18.4%, and 15.8%, respectively; the soil organic carbon, total nitrogen, total carbon, silt, and clay contents increased by 39.5%, 31.3%, 30.7%, 70.8%, and 61.3%, respectively; the vegetation-soil system coupling also increased; while the sand contents decreased by 20.7%. Therefore, in the Ulan Buh Desert, we recommend two replanting species and their densities: *C. korshinskii* with 18-44 individuals per 100 m^-2^, and *H. ammodendron* with 24-30 individuals per 100 m^-2^ (**Table 2**; **Supplementary Table S1**).

In the Kubuqi Desert, compared to the natural *A. ordosica* community, the herbaceous abundance, woody abundance, herbaceous coverage, and woody coverage of the revegetated *A. ordosica* community increased by 3.63%, 72.2%, 18.6%, 58.5%, respectively; the soil organic carbon, total nitrogen, total carbon, total phosphorus, silt, and clay contents increased by 57.7%, 27.6%, 10.7%, 21.7%, 27.7%, and 22.5%, respectively; the vegetation-soil system coupling also increased; while the sand content decreased by 4.83%. As a result, in the Kubuqi Desert, we recommend two replanting species and their densities: *A. ordosica* with 60-70 individuals per 100 m^-2^ and *C. korshinskii* with 20-45 individuals per 100 m^-2^ (**Table 2**; **Supplementary Table S1**).

## 4 Discussion

### 4.1 The selection and establishment of revegetation ameliorate the conditions for plant survival

Consistent with our first hypothesis, plant and soil conditions improved compared to those of the moving sand dunes and became similar to those in natural vegetation areas following revegetation. This study finds that herbaceous species diversity significantly increased comparing to adjacent natural vegetation communities after the establishment of revegetation in the Ulan Buh Desert, whereas herbaceous biomass, woody richness, and soil water content significantly decreased. This may be due to shrub planting effectively stabilizing the surface of sandy soil and improving soil nutrient conditions, thus providing a suitable environment for the settlement and germination of herbaceous plant seeds (Angers and Caron,1998; Miao et al., 2015; Zhong et al., 2018). Meanwhile, the root turnover of woody plants also provides essential nutrients for the growth of herbaceous plants. Therefore, the abundance, richness, and diversity indices of herbaceous plants have increased (An, 2019). However, the growth of woody species also competed for some soil nutrients and water resources, leading to the reduction in the herbaceous biomass and woody richness (Rossatto et al., 2014). In addition, the root water uptake of woody vegetation resulted in the decline soil water content (Pellissier et al., 2008). The establishment of revegetation in the Kubuqi Desert, herbaceous indicators, soil nutrients, silt, and clay contents all declined when compared to natural vegetation communities, whereas woody abundance increased. These might be illustrated that the introduction of woody vegetation increased the abundance of woody species. Meanwhile, the competition for nutrients and water among these woody plants was relatively intensive, attenuating the resource availability and thus inhibiting the growth of original herbaceous plants (Cui et al., 2019; Zheng, 2022).

In the present study, we found that the establishment of revegetation significantly increased the diversity of herbaceous plants, biomass, and soil nutrients, which is attributed to the woody vegetation creating more suitable growth conditions for herbaceous plants and increasing the content of silt and clay in the soil, thereby improving soil structure and stability (Ou et al., 2020; Scotton and Andreatta, 2021; Kumar et al., 2022). Vegetation restoration enhanced soil organic matter content through the input of roots and residues, which in turn increased soil fertility, beneficial for the growth of woody plants, and reduced wind erosion, providing a more stable growth environment for plants and promoting their reproduction and spread (Lajtha et al., 2014; Mitchell et al., 2018; Zhang et al., 2020; Zhu et al., 2010; Mensah, 2015; Maiti et al., 2021; Luo et al., 2020). Meanwhile, vegetation restoration improved the physical, chemical, and biological characteristics of the soil, increased soil nutrient content and water retention capacity, and enhanced species diversity (Xu et al., 2010; Chen et al., 2023; Zhou et al., 2023; Sheoran et al., 2010; Ploughe et al., 2021). In the early stages of vegetation restoration, effective soil coverage reduced water evaporation and soil erosion, helping to maintain soil nutrients (Li et al., 2004; Zhang et al., 2018). Overall, the vegetation subsystem, soil subsystem, and the vegetation-soil system coupling all increased after restoration, and these improvements collectively promoted the favorable status and interaction of the vegetation and soil systems (Li and Liber, 2018; Guo et al., 2021).

Our findings provided scientific evidence for windbreak and sand-stabilization in these two deserts, and gave important guidance to future vegetation restoration in deserts. Traditionally, the success of restoration was determined by monitoring plant cover, individual counts, and soil characteristics after revegetation (Cudjoe, 2011; Song, 2018) as well as by selecting suitable species by greenhouse, field experiments, and seedling survival tests (Jusaitis and Pillman, 1997; Gastauer et al., 2020). Here, we considered not only the vegetation and soil systems, but also the vegetation-soil coupling in different vegetation types to select the most suitable revegetation strategy.

### 4.2 Coupling coordination of vegetation-soil system in two deserts

Consistent with our second hypothesis, we found that different community types exhibited variations in the vegetation-soil system coupling in these two deserts, which indicted that species selection was extremely important for the sustainability ecological restoration in deserts. The consistence of the changes between vegetation and soil indicators elucidated that plant growth and soil properties were closely correlated. There is a feedback mechanism between vegetation and soil. The plants exert a huge influence on soil conditions, which in turn support plant growth (Bever et al., 1997; Ehrenfeld et al., 2005; Miki, 2012; Maiti and Ghosh, 2020). Vegetation alters soil properties through physical and chemical processes during the restoration process (Cao et al., 2008; Ghestem et al., 2011; Wu et al., 2019). Meanwhile, vegetation roots increase soil porosity, and the decomposition of plant material adds organic matter to soil (Wu et al., 2016). In a word, different community types have varying impacts on soil properties, leading to disparities in the vegetation-soil systems coupling of different community types. The improvements of soil properties and vegetation growth enhance soil aggregate stability and resistance to wind erosion (Dou et al., 2020), thereby creating a positive feedback loop within the vegetation-soil system.

In this study, we found that soil nutrient content, herbaceous and woody indicators were positively correlated with the coupling degree of the vegetation-soil system. These were consistent with our hypothesis that the vegetation-soil system coupling was mainly affected by vegetation and soil characteristics. Generally, superior soil conditions promote plant growth, which in turn improves soil structure and nutrient cycling, thus forming a positive feedback loop. The mutual promotion and dependence between vegetation and soil constituted a well-coupled ecosystem, demonstrating a positive coupling state between vegetation-soil systems (Van der Putten et al., 2013; Hobbie, 2015; Lehman et al., 2015; Wang et al., 2020; Aqeel et al., 2023). Simultaneously, this feedback loop suggests that healthy soil and abundant vegetation maintain ecosystem stability and functionality (Powlson et al., 2011; Mensah, 2015). Our findings indicated that unsuitable soil pH, high sand content, high wind speed, and high temperature adversely influenced plant growth and soil conditions (Zuo et al., 2012). These factors can also lead to soil degradation, nutrient loss, and reduce water availability, which in turn reduce plant growth and survival. The plant degeneration subsequently reduced vegetation cover and richness, exacerbating soil degradation (Duniway et al., 2019; Huang and Hartemink, 2020; Zhang et al., 2021; Ferreira et al., 2022). Taken together, coupling coordination of vegetation-soil system is crucial for maintaining the stability of ecosystems after the establishment of revegetation, as it directly affects the normal functioning of ecological processes and the long-term health of the ecosystem.

## 5 Conclusion

In our study, revegetation significantly improved the vegetation and soil conditions in the Ulan Buh Desert and Kubuqi Desert of Northern China. Water conditions, vegetation growth, and soil nutrients all influenced the vegetation-soil system coupling. We propose a method could be used for evaluating the effects of different revegetation types on the vegetation subsystem, soil subsystem, and the vegetation-soil system coupling. This method can select optimum plant species and its density, as well as evaluate the effects of revegetation on global desertification prevention. Our findings provide important guidance for vegetation reconstruction and ecological restoration in deserts.

## Data Availability

The raw data supporting the conclusions of this article will be made available by the authors, without undue reservation.
