# Supplementary material for: Coupling relationships between vegetation and soil in different vegetation types in the Ulan Buh Desert and the Kubuqi Desert
Source: Front Plant Sci. 2025 Mar 13;16:1505526. doi: 10.3389/fpls.2025.1505526 (PMC11966041; doi:10.3389/fpls.2025.1505526)
Supplement: Supplementary file 1 [file DataSheet1.docx]

Supplementary Material

# Supplementary Figures and Tables

## Supplementary Figures

**
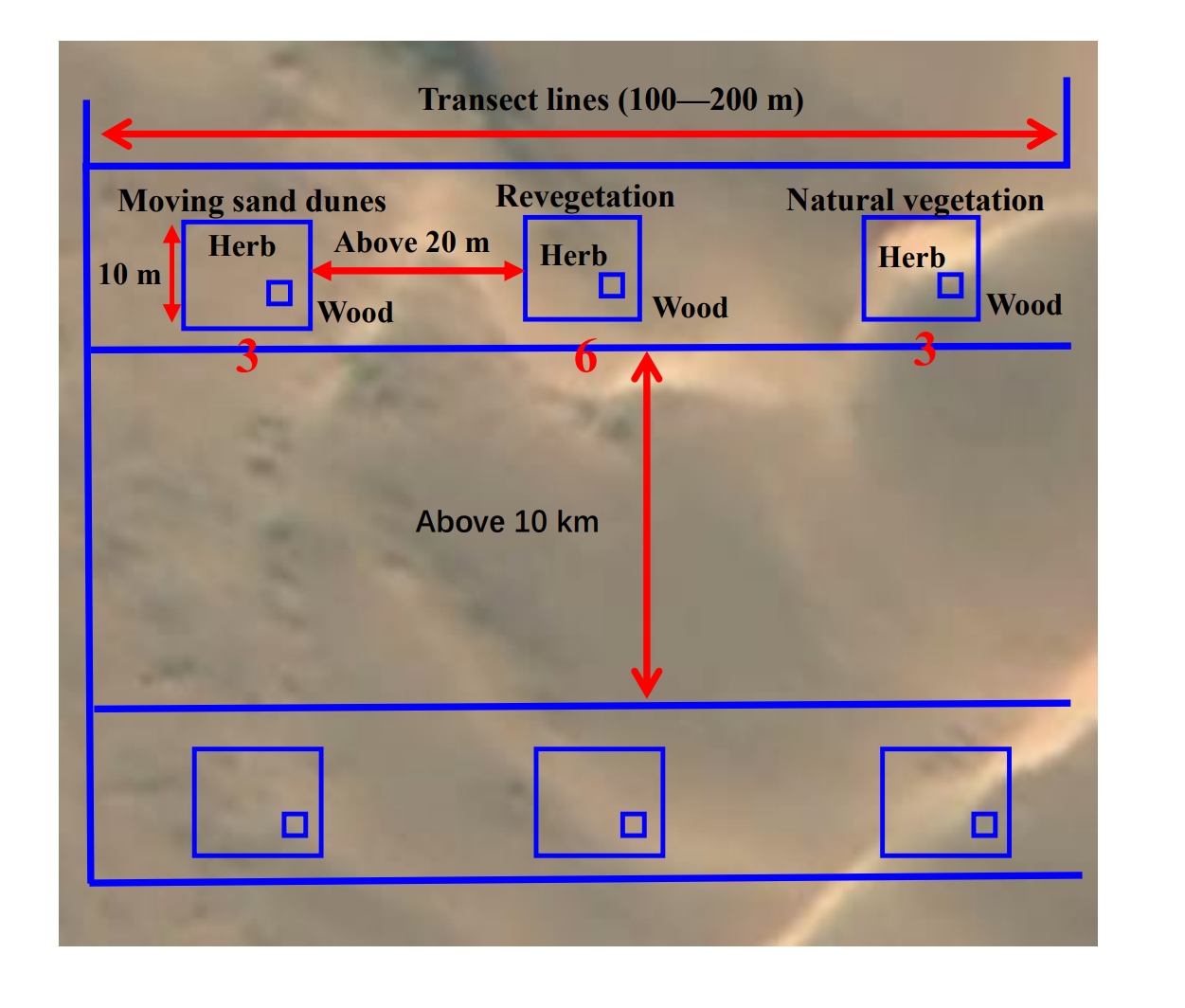
**

**Supplementary Figure 1.** Quadrats setting diagram

**Supplementary Figure 2.** Correlation matrix between vegetation and soil indexes

Notes: *indicates significant correlation (*p*<0.05), and ** indicates highly significant correlation (*p*<0.01), and *** indicates highly significant correlation (*p*<0.001). HA:Herbaceous abundance; HR:Herbaceous richness; HSH:Herbaceous Shannon-Wiener index; HSI:Herbaceous Simpson index; HC:Herbaceous coverage(%); HB:Herbaceous biomass(g m^-2^); WC:Woody coverage(%); WA:Woody abundance; WR:Woody richness; WSH:Woody Shannon-Wiener index; WSI:Woody Simpson index; SWC:Soil water content (%); BUD:Bulk density(g cm^-3^); SAW:Saturated water content ; OC:Organic carbon(g kg^-1^); TN:Total nitrogen(g kg^-1^); TC:Total carbon(g kg^-1^); TP:Total phosphorus(g kg^-1^); Cond:Conductivity (us cm^-1^); SAN:Sand(%); SIL:Silt(%); CLA:Clay(%)

## Supplementary Table

**Supplementary Table 1.** Vegetation indices and soil indices of different community types in the Ulan Buh Desert and Kubuqi Desert

| Desert | Area | Community category | Number | Herbaceous abundance | Herbaceous richness | Herbaceous coverage (%) | Woody coverage (%) | Woody abundance | Woody richness | Initial density (individuals /100 m^2^) | Organic carbon (g kg^-1^) | Total nitrogen (g kg^-1^) | Total Carbon (g kg^-1^) | Total phosphorus (g kg^-1^) | Sand (%) | Silt  (%) | Clay (%) |
| --- | --- | --- | --- | --- | --- | --- | --- | --- | --- | --- | --- | --- | --- | --- | --- | --- | --- |
| Ulan Buh Desert | Natural | Pha | 3 | 4.7±1.86bc | 1±0.89bc | 23±12.4a | 0 c | 0 b | 0 e | / | 0.3±0.03bc | 0.2±0.02cd | 1.4±0.03b | 0.4±0.01bc | 92±6.36abc | 0.4±0.05d | 7.4±3.41abc |
|  |  | Nit | 9 | 3.4±1.11bc | 0.9±0.20c | 13±5.04ab | 12±3.43bc | 5±1.05b | 1.8±0.36cd | / | 1.1±0.24bc | 0.3±0.06bc | 6.5±2.23ab | 0.7±0.14ab | 71±10.8bc | 17±7.50abc | 12±4.08ab |
|  |  | Kaf | 9 | 5.6±2.27abc | 0.7±0.24c | 4.4±1.81b | 27±5.11b | 229±51ab | 2.2±0.22abcd | / | 3.2±0.35a | 0.5±0.04a | 9.3±1.60a | 0.9±0.06a | 68±4.48c | 18±2.86ab | 14±1.76a |
|  |  | Amm | 3 | 1.7±0.67bc | 1.7±0.67bc | 4.4±0.41b | 11±1.37bc | 12±0.58b | 3.3±0.33a | / | 1.1±0.05b | 0.4±0.02ab | 6.3±0.62ab | 0.4±0.04bc | 85±4.46abc | 7.6±2.01abcd | 7.4±2.51abc |
|  |  | Aro | 15 | 4.3±1.11bc | 1.6±0.25bc | 5.6±0.92b | 14±3.38bc | 179±41a | 1.6±0.21d | / | 0.6±0.06bc | 0.3±0.03bcd | 4.3±0.34ab | 0.4±0.02bc | 91±2.55abc | 5.9±2.27bcd | 3.1±0.49bc |
|  |  | Cos | 3 | 2.7±0.88bc | 1.3±0.33bc | 6±1.15ab | 55±13.1a | 7±1.20b | 2.7±0.33abc | / | 0.5±0.03bc | 0.2±0.03cd | 3.8±0.05b | 0.5±0.01bc | 96±0.21a | 1.6±0.04cd | 2.4±0.18c |
|  |  | Aro-Ars | 3 | 3±0.58bc | 1 bc | 8±2.65ab | 3.1±0.7c | 28±10.5b | 2 bcd | / | 0.3±0.02c | 0.1±0.02d | 1.6±0.05b | 0.3±0.01c | 98±0.21a | 0.7±0.12cd | 1.3±0.10c |
|  |  | Nit-Haa | 9 | 4.8±1.61bc | 1.3±0.29bc | 9.4±4.27ab | 20±8.20bc | 13±3.72b | 2.9±0.46abc | / | 0.7±0.05bc | 0.3±0.02bcd | 3.6±0.51b | 0.7±0.08ab | 86±3.11abc | 6.6±2.23bcd | 7.6±1.31abc |
|  |  | Cak | 3 | 12±0.58 a | 4.3±0.33a | 17±2.96ab | 13±1.33bc | 11±2.08b | 2±0.66bcd | / | 0.4±0.06bc | 0.2±0.02cd | 2.4±0.04b | 0.5±0.01bc | 92±2.77abc | 5.5±2.59bcd | 2.5±0.80bc |
|  | Revegetation | Haa | 24 | 6.9±1.54abc | 2±0.18b | 9.9±2.17ab | 14±2.72bc | 15±1.54b | 2.2±0.10abcd | 24-30 | 0.8±0.11bc | 0.3±0.01bc | 5.4±0.28ab | 0.5±0.01bc | 93±1.59abc | 3.2±0.77bcd | 3.8±0.86abc |
|  |  | Cos | 3 | 3.7±1.76bc | 1 bc | 12±4.98ab | 6±0.52c | 15±2.65b | 3 ab | 12 | 0.4±.08bc | 0.1±0.02d | 2.3±0.21b | 0.4±0.01bc | 93±1.09ab | 1.2±0.09cd | 5.8±0.99abc |
|  |  | Cak | 6 | 7.7±2.9 ab | 2.7±0.21bc | 6.2±1.25ab | 14±3.02bc | 12±1.47b | 3±0.26ab | 18-44 | 0.7±0.06bc | 0.3±0.02bcd | 3.4±0.12b | 0.3±0.04c | 73±2.17bc | 23±4.68a | 6±2.52abc |
|  | Moving sand dunes | Moving sand dunes | 9 | 0.2±0.15c | 0.2±0.15c | 1.4±1.32b | 0 c | 0 b | 0 e | / | 0.4±0.02bc | 0.2±0.02cd | 2.7±1.16b | 0.4±0.02bc | 98±0.14a | 0.4±0.09d | 1.6±0.08c |
| Kubuqi Desert | Natural | Aro-Nit | 3 | 6.3±0.88e | 2.7±0.33cde | 20±5.24defg | 33±25.2abcdef | 26±0.08cd | 2 def | / | 1.4±.26def | 0.5±0.09cde | 7.8±1.85bc | 0.6±0.07bc | 76±3.54h | 10±2.19ab | 14±0.86a |
|  |  | Aro | 24 | 48±9.92bcd | 3.9±0.41bc | 51±4.79abc | 21±4.74bcdef | 42±6.35bcd | 1.7±0.14cdef | / | 2.0±0.36cdef | 0.5±0.05def | 5.6±0.39cde | 0.7±0.02b | 91±1.46bcd | 4.7±0.94cd | 4.3±0.57efg |
|  |  | Krc | 3 | 8±2.52e | 3.7±0.34bc | 25±5.03cdefg | 9±0.64ef | 34±3.18cd | 3.3±0.33bc | / | 2.9±0.12bc | 0.8±0.05a | 6.6±0.72cd | 0.5±0.01cd | 80±2.53gh | 10±1.86a | 10±0.67b |
|  |  | Cab | 3 | 70±7.22ab | 6 a | 37±7.05bcdef | 6±0.83f | 108±42ab | 3 bcd | / | 2.6±0.18bcd | 0.7±0.03ab | 4.5±0.44def | 0.4±0.01def | 88±2.42de | 6.1±0.96c | 5.9±1.46cde |
|  |  | Cat | 3 | 81±12.2a | 2.7±0.33cde | 56±8.45ab | 9±0.64ef | 53±14.9bcd | 3.3±0.33ab | / | 3.2±0.14ab | 0.7±0.02a | 5.8±0.03cd | 0.6±0.01bc | 82±0.65fg | 12±0.43a | 7.3±0.34bcd |
|  |  | Nit-Tac | 3 | 77±20.4a | 3.7±0.33bc | 68±6.98a | 19±1.55cdef | 3.3±0.33d | 2 a | / | 4.5±0.39a | 0.8±0.06a | 11±0.56a | 0.8±0.04a | 89±1.02cde | 5.5±0.48c | 5.5±0.54def |
|  |  | Nes | 3 | 26±5.29cde | 5.7±0.33a | 51±7.88ab | 53±19.1ab | 155±31a | 2±0.58cde | / | 1.8±0.50cde | 0.6±0.05bcd | 9.1±1.24ab | 0.6±0.04bc | 86±2.36ef | 7.5±1.05c | 6.5±1.41bc |
|  |  | Aro-Cof | 9 | 56±9.50bc | 3.3±0.62cdef | 47±9.74ab | 54±6.35abc | 86±5.5bc | 2.9±0.39ef | / | 1.4±0.34ef | 0.3±0.05gh | 3.6±0.65dfg | 0.5±0.04cde | 97±0.33a | 1.5±0.19de | 1.5±0.23gh |
|  |  | Cof | 3 | 28±3.28cde | 3±0.58bcd | 43±1.53bcd | 22±8.96bcdef | 18±7.55cd | 3±0.58def | / | 1.3±0.84 def | 0.3±0.08fgh | 4.7±1.09def | 0.6±0.06bc | 97±0.57a | 1.3±0.55de | 1.4±0.13gh |
|  | Revegetation | Cof | 24 | 23±4.28cde | 2.4±0.15cdef | 34±2.88bcdef | 43±4.47abcd | 30±3.90cd | 3.3±0.22ef | 21-40 | 0.6±0.07ef | 0.2±0.01gh | 3.1±0.22efg | 0.5±0.01cd | 98±0.07a | 0.8±0.09e | 1.4±0.10gh |
|  |  | Haa | 6 | 3.5±0.76e | 1.2±0.17ef | 4.5±1.54g | 11±2.29def | 75±19.9ab | 2 ef | 33 | 0.7±0.08ef | 0.2±0.03fgh | 4.2±0.32defg | 0.4±0.01ef | 88±1.18de | 7.1±0.66c | 4.9±0.52def |
|  |  | Cam | 6 | 20±5.39de | 3±0.37bcd | 37±6.68bcde | 8±1.34f | 18±3.17cd | 2.5±0.43f | / | 0.3±0.03f | 0.3±0.01fgh | 1.9±0.05g | 0.4±0.01ef | 98±0.30a | 1.2±0.19de | 0.8±0.11h |
|  |  | Sac | 32 | 21±3.89de | 2.4±0.19def | 22±3.20defg | 31±3.57abcdef | 57±13.6bcd | 2.8±0.20ef | 18-44 | 0.6±0.07ef | 0.3±0.02gh | 3.2±0.24efg | 0.5±0.02cde | 98±0.12a | 0.9±0.07de | 1.5±0.09gh |
|  |  | Aro | 9 | 50±10.1abc | 3.7±0.21bcd | 61±7.50ab | 49±13.0a | 133±41a | 1.5±0.22a | 60-70 | 4.4±1.18a | 0.7±0.06abc | 6.4±0.31cd | 0.9±0.02a | 87±0.99de | 7.4±0.40bc | 5.7±0.58cde |
|  |  | Pop | 15 | 33±5.32cde | 3.1±0.29cd | 27±4.22cdefg | 25±4.63bcdef | 64±7.57bcd | 2.5±0.22def | 14 | 1.3±0.40def | 0.3±0.03gh | 2.4±0.38fg | 0.5±0.03cdef | 97±0.42a | 1.5±0.25de | 1.5±0.12gh |
|  |  | Pop-Sac | 6 | 3.2±0.87e | 1.3±0.21ef | 0.2±0.04g | 23±8.38abcdef | 21±6.91cd | 1.8±0.31f | 31 | 0.3±0.02f | 0.2±0.01h | 1.9±0.25g | 0.1±0.03f | 97±0.66a | 0.7±0.15e | 2.2±0.60gh |
|  |  | Cak | 6 | 54±9.02abc | 4.3±0.33b | 16±4.33efg | 41±7.01abcde | 65±7.86bcd | 2.3±0.33def | 20-45 | 1.3±0.42def | 0.4±0.03efg | 10±1.29ab | 0.7±0.02b | 94±1.46abc | 1.9±0.53de | 3.8±0.94efg |
|  | Moving sand dunes | Moving sand dunes | 27 | 3.2±0.75e | 1.2±0.16f | 11±2.14fg | 3±1.55f | 3±1.55d | 0.7±0.27ef | / | 0.4±0.03ef | 0.2±0.01h | 2.9±0.40efg | 0.5±0.03cd | 96±1.04ab | 0.7±0.07e | 2.6±1.04fgh |

Notes: The mean value in the table is the mean ± standard error (SE). Different lowercase letters indicate significant differences in different vegetation types (α = 0.05).

Pha:*Phragmites australis*; Nit:*Nitraria tangutorum*; Kaf:*Kalidium foliatum*; Amm:*Ammopiptanthus mongolicus*; Aro:*Artemisia ordosica*; Cos:*Corethrodendron scoparium*; Ars:*Artemisia sieversiana*; Haa:*Haloxylon ammodendron*; Cak:*Caragana korshinskii*; Krc:*Krascheninnikovia ceratoides*; Cab:*Caragana brachypoda*; Cat:*Caragana tibetica*; Tac:*Tamarix chinensis*; Nes:*Neotrinia splendens*; Cof:*Corethrodendron fruticosum*; Cam:*Calligonum mongolicum*; Sac:*Salix cheilophila*; Pop:*Populus przewalskii*
